# The Medical Curriculum

**Published:** 1920-07-10

**Authors:** 


### The Medical Curriculum.

ONE of the most eager of the sectional discussions during the meeting of the British Medical Association at Cambridge was on the subject of the medical curriculum. It was presided over by Sir George Newman, who devoted a large part of his introductory address to the question of the clinical teaching unit. There was nothing celestial, he said, about such a unit. It was merely a matter of convenient arrangement by which three general advantages were secured: (1) The clinical teacher devoted a regular and substantial portion of his time to his teaching work, which became his chief task, and for the student, instruction in clinical medicine and surgery was thus systematised, thorough, and always available; (2) the unit consisted of a staff of competent men working as a group or team and pooling their experience—the physician, the assistant physician, the resident physician, and the house physician; (3) there was full integration of the science and art of medicine and surgery, the teaching of which might thus be raised to university standard. Sir George cited examples from London and Edinburgh. At University College, London, Mr. Choyce had been appointed whole-time director of surgery, with two whole-time assistants, and with forty beds, out-patients, laboratory accommodation, and special departments. The work of the unit consisted of routine clinical work, teaching, pathological investigation and research, recording cases, and certain special branches of surgery. The arrangements for teaching included clinical demonstrations in the wards, supervision of dressers, systematic lectures on surgery, operative surgery, surgical pathology, practical and clinical surgery, and a surgical revision class, and the unit co-operated with the extra-unit clinics of the hospital. The advantage to the students was that their surgical teachers were always available, and thus they secured personal and continuous tuition. They saw surgery as a whole, and discovered a complete integration of its science and art, its essential integrity and unity. In varying degree, this kind of teaching was becoming the usual practice in the best medical schools. It was bringing with it closer study, larger vision, wider comprehension, a more intimate co-ordination of all branches of medicine, and a deeper unity. The reforms in the curriculum which Sir George advised were four: (1) A lightening at both ends: in other words, fuller preparation in science before entrance to the medical school, and a postponement of instruction in certain specialities to the post-graduate period; (2) a fuller study of the sciences preliminary to medicine; (3) the development of clinical teaching of university standard; (4) further State aid, though with a minimum of State control.

Among the several papers which followed Sir George Newman's address, one of the most remarkable was forthcoming from Professor Keith. One passage of his may be quoted. He held that no man was fit to teach medical students unless he himself was a qualified practitioner of medicine and maintained his knowledge in a state which would permit him to ply his real vocation. If there was to be a compulsory training for the medical practitioner, why not also a compulsory training for the medical teacher? Was it too much to expect that he should be able to scrape a bare pass in the examination papers which his colleagues set for students? At great medical schools every teaching anatomist and physiologist should have to hold occasional clinical appointments up to his thirty-fifth year.

One of the most interesting contributions to the subsequent discussion was made by Professor Giacosa, of Turin. He agreed with Sir George Newman as to the necessity for a sound scientific basis for the coming physician, who ought not to be able to announce himself a specialist in any branch unless he had been through the fundamental curriculum. The specialist ought in every case to follow post-graduate courses and to go through special examinations. That was what was going to be established in Italy.

Three resolutions were placed in the hands of the President of the section (Sir George Newman), but they were not formally put to the meeting, which concurred in Sir George Newman's offer to transmit them, along with the full text of the discussion, to the Educational Grants Board, of which he was an assessor, and to the Education Committee of the General Medical Council. One of the resolutions was that the lowest age of registration for a medical student should be seventeen years; the second, that in the forthcoming revision of curriculum promised by the General Medical Council the lightening of professional studies would be facilitated if chemistry and physics were made school subjects; and the third, that a knowledge of physics and chemistry of matriculation standard should be exacted of all students before they registered.

